# Misreported Cancer Deaths in PLATO Trial

**DOI:** 10.3390/jcm10143140

**Published:** 2021-07-16

**Authors:** Victor Serebruany, Jean-Francois Tanguay

**Affiliations:** 1 Department of Neurology, Johns Hopkins University, Baltimore, MD 21218, USA; 2 Montreal Heart Institute, Université de Montréal, Montreal, QC H1T 1C8, Canada; jean-francois.tanguay@icm-mhi.org

**Keywords:** clinical trial, ticagrelor, clopidogrel, death, cancer

## Abstract

The potential link between antiplatelet agents and anticoagulants with excess cancer deaths (CD) was reported first for prasugrel (TRITON, DAPT), clopidogrel (DAPT), vorapaxar (TRACER), apixaban (APPRAISE-2), and later ticagrelor (PEGASUS). However, verified CD in the ticagrelor indication-seeking PLATO were not public. We obtained the complete list of deaths and their primary causes in PLATO, matched that dataset against local site records, and analyzed the patterns of CD reporting. The FDA-issued spreadsheet contains 31 precisely detailed CD (PLATO code 12-3). We obtained local site evidence for four CD and matched them with FDA-reported. We also assessed the patterns of how CD were reported among non-vascular death database column “S” by scrolling the FDA Excel file down among 938 PLATO entries. Clopidogrel CD (*n* = 17) were reported exclusively by sponsor, while independent CRO’s reported only ticagrelor CD (3 out of 14 PLATO total). Among four matched verified outcomes, one ticagrelor CD was correct, second ticagrelor CD was unreported, and two (ticagrelor and clopidogrel) CD were reported inaccurately. Of the remaining 16 clopidogrel CD six were reported as three separate next in line paired entries in Denmark (236–237), Poland (597–598), Romania (679–680), and as two more fatalities in South Africa (786) and Spain (789), while patients 787 and 788 received ticagrelor out of 938 records suggesting possible late addition of incorrect clopidogrel CD reports. We conclude that some CD were misreported in PLATO, favoring ticagrelor. Such mismatch may require reevaluation of this critical outcome in the trial focusing on the exact death cause reported by site investigators.

## 1. Introduction

**Figure 1:**
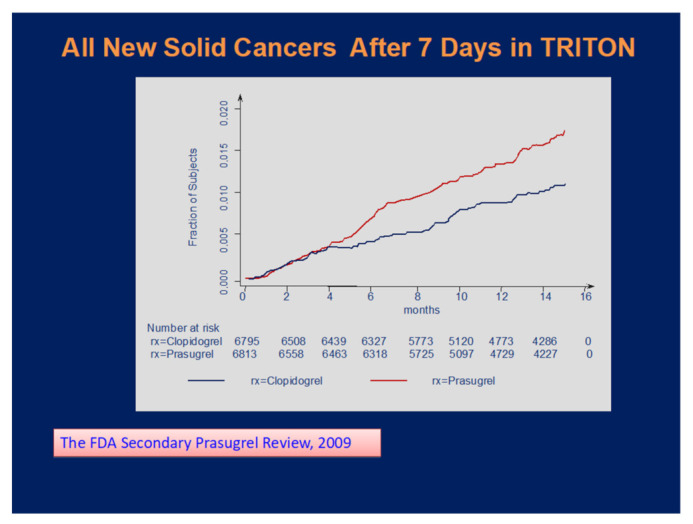
Incidence of new solid cancers in TRITON trial.

The link between malignancy and hemostasis is well-established, contributing to inflammation, atherosclerosis, and cancer dissemination through the release of cytokines, chemokines, and expression of several adhesion receptors [1]. The first alarming signal that potent antiplatelet therapy may be associated with an excess of solid cancers was observed in the prasugrel arm of TRITON-TIMI 38 trial [2,3]. The curves are exhibited in Figure 1.

Later evidence from randomized trials remains inconclusive, possibly due to low incidence, or missed precise outcomes. In the PLATO trial ticagrelor lead to a significant reduction in the primary endpoint (a composite of death from vascular causes, myocardial infarction, or stroke) compared to clopidogrel (9.8% vs. 11.7%, 95% CI; 0.77–0.92, *p* < 0.001) [4]. The trial investigators reported more neoplasms after clopidogrel (155 vs. 132 cases), however, the cancer deaths were missing [4]. We finally gained access to the detailed FDA-issued dataset of PLATO deaths, which has been matched with local patient-level data from sites controlled by the sponsor, revealing that the actual existence, precise dates, and proper causes of some deaths in PLATO were inaccurately reported in favor of ticagrelor [5]. Moreover, there is a significant discrepancy between primary death causes reported to the FDA, and those utilized by the PLATO for numerous secondary reports published in top journals for over a decade [6]. We here examined the validity and reporting patterns of cancer deaths in PLATO.

## 2. Methods

Based on the Freedom of Information Act, BuzzFeed filed a legal complaint in the US Federal Court, won an expedited order, and shared with us the complete PLATO death list submitted to the FDA by the ticagrelor NDA 22-433 sponsor. The FDA spreadsheet contains 938 PLATO deaths with trial ID numbers, country, enrolling site, patient age, gender, treatment assignments, discontinuations, outcome codes, dates, and precise causes of trial exit. Each event contains whether the death cause was vascular (code 11), non-vascular (code 12), or unknown (code 97). There were 14 subcodes for vascular, 9 subcodes for non-vascular deaths, and universal code “99” which applied for “other” causes. Most of the data were controlled and reported by PLATO sponsor, with the exception of USA, Russia, Georgia, and most (sites 5101–5106) of Ukraine. The entire US was monitored by ReSearch Pharmaceutical Services (Wort Washington, PA, USA; http://www.rpsweb.com (accessed on 14 July 2021)). All Russian, Georgian, and most Ukrainian sites were monitored by Evidence CRP, now Worldwide Clinical Trials, (Morrisville, NC, USA; http://wwctrials.com/ (accessed on 14 July 2021)). The FDA-issued list contains 31 precisely detailed cancer deaths for which the primary cause was non-vascular “12”, and cancer “3” for the unique PLATO code “12-3”. We examined local records on 4 of such deaths among 873 PLATO patients with 53 deaths from 15 enrolling sites in 8 countries and matched those with what was reported to the FDA. We also assessed the reporting pattern of cancer deaths issued by the FDA just scrolling column “S” the non-vascular death causes.

## 3. Results

Among 31 FDA-reported cancer deaths in PLATO, those attributed to clopidogrel (*n* = 17) were numerically more than after ticagrelor (*n* = 14). However, all clopidogrel cancer deaths were reported exclusively by the PLATO sponsor, while independent CRO’s reported only ticagrelor cancer deaths (*n* = 3). We matched four PLATO deceased patient records (three ticagrelor and one clopidogrel) with local site data. One ticagrelor cancer death was reported correctly, a second ticagrelor cancer death was unreported, and two (ticagrelor and clopidogrel) cancer deaths were reported inaccurately. Indeed, in one case from Hungary, the cause of death was truly non vascular (code 12), but not from cancer (subcode 3). As reported by site, the primary cause of death of that particular clopidogrel patient was respiratory failure (subcode 1). Another interesting case was reported in Canada. With regard to the primary death cause, that ticagrelor patient experienced a fatal bleed (twice 2L of hemorrhagic pleural effusion four days apart). On first drainage, adenocarcinoma cells of unknown origin were found in the pathologist report. The next day, this patient experienced severe bradycardia, cardiac arrest with asystole that could not be reanimated. His hemoglobin went from 12.5–13.1 to 9.3 g/L. The medical summary report clearly indicated “cardiopulmonary failure secondary to metastatic adenocarcinoma of unknown origin”. In short, either fatal bleeding or cardiopulmonary failure were true causes of death, but both causes were considered as “vascular” per PLATO protocol, and switched to cancer (non-vascular) in ticagrelor patient. Of the remaining 16 clopidogrel cancer deaths, half were reported as three separate pairs doubling next patient record and two more consecutive entries, suggesting possible last-minute addition of incorrect cases and post-monitoring database manipulation. See Table 1 for details. 

The surprising pattern of these pairs of next in line clopidogrel patients marked as cancer death can be easily detected by just scrolling down Excel list column “S” indicative of non-vascular death primary cause. Repeated placement in pairs of subcode 3 (cancer) for eight clopidogrel patients could strongly suggest possible database manipulation that artificially worsened clopidogrel risks.

## 4. Discussion

The main finding of this report suggests that ticagrelor is not better than any other antithrombotics, when prolonged and aggressive strategies indeed may be associated with increased cancer risks. Aside from the misreporting possibility, the claims that ticagrelor represents a “success” for cancer in PLATO appear incorrect. The drug per se probably does not cause a direct carcinogenic effect but contributes negatively via excessive platelet inhibition when used long-term at full dose. Assessing cancer signals after ticagrelor was challenging since the first reliable evidence was obtained from the second trial (PEGASUS) when significantly more cancer deaths (77 vs. 53; RR = 1.46; 95%CI 1.02–2.06, *p* = 0.034) were reported after ticagrelor than in the placebo arm [7]. The PEGASUS data are in full agreement with the DAPT trial [8], suggesting, similar to prasugrel, extra cancer risks after ticagrelor beyond one-year therapy. In contrast to the balanced and mildly concerned FDA report [9], the official PLATO publication overoptimistically presents the cancer data as somewhat an “advantage” of ticagrelor. Any new neoplasms during PLATO were 132 vs. 155 (*p* = 0.17), including malignant (115 vs. 121, *p* = 0.69), and benign (18 vs. 35; *p* = 0.02) all in favor for ticagrelor [4]. The problem with these numbers is two-fold. First, in a short trial with incomplete vital status follow-up in 531 PLATO patients [2], the cancer data are difficult to evaluate, especially when the study sponsor self-monitored over 80% of sites. Second, the historical evidence indicates that within one year follow up, clopidogrel cancer rates are not higher than those yielded on aspirin. Since all ticagrelor patients in PLATO received aspirin as well, which caused more bleeding than clopidogrel, there is no biological plausibility for fewer cancer risks after ticagrelor, especially considering positive animal studies. The cancer signal after prasugrel in TRITON was an unexpected finding, therefore, it will be important to obtain complete follow-up in later trials, which was lacking in PLATO [9,10].

It is of interest to note that in ticagrelor preclinical carcinogenicity studies, while experiments in mice were negative, a ticagrelor dose-escalating study on rats demonstrated a significant decrease in female survival (Cox: *p* = 0.018, Kruskal–Wallis: *p* = 0.042) due to metastatic uterine neoplasms, mostly adenocarcinomas, and squamous cell carcinomas. Fourteen of the 31 female animals in high-dose ticagrelor who died ahead of scheduled termination had uterine adenocarcinoma listed as the cause of death. Such increased incidence of mortality was statistically significant when compared to the combined controls (*p* < 0.001). It has also been proposed that changes in circulating prolactin may cause animal tumor findings [9]. Moreover, an excess of gynecomastia [4] in the ticagrelor PLATO arm (0.19% vs. 0.03%, RR = 6.0) suggests prolactin involvement, and estrogen burst to increase the size of male breast tissue [9]. The submitted data suggest no evidence for ticagrelor effects upon rates of sex organ malignancies, although PLATO was a relatively short study with only 277 days of mean follow-up [4,9,10].

Some cancer deaths misreporting in PLATO will require a reevaluation since a mismatch of death dates, and especially their causes were already reported [5]. The possible incorrect cancer death reporting for four pairs of eight clopidogrel “cancer” deaths remains to be elucidated. Such an investigation is not a small task and could remain a major challenge for independent researchers without having access to this critical trial dataset. In the end, the data suggest that cancer deaths may have been underreported as a primary cause in PLATO, with only 3.3% fatalities, including these four pairs of questionable clopidogrel deaths. 

## 5. Conclusions

Several cancer deaths were misreported in PLATO, favoring ticagrelor. After the FDA discovered the cancer signal associated with prasugrel in TRITON, all efforts should be made to properly elucidate this critical outcome in PLATO.

## Figures and Tables

**Table 1:** Questionable cancer deaths in PLATO trial.

ENTR	Age	Gender	STUDYDY	Country	TRTRTXT	NVASSCLS
236	69	Male	9	Denmark	Clopidogrel	3
237	84	Male	7	Denmark	Clopidogrel	3
597	68	Male	400	Poland	Clopidogrel	3
598	52	Male	157	Poland	Clopidogrel	3
679	85	Female	302	Romania	Clopidogrel	3
680	60	Male	191	Romania	Clopidogrel	3
786	69	Male	227	S. Africa	Clopidogrel	3
789 **	80	Male	327	Spain	Clopidogrel	3

ENTR—patient number among 938 reported PLATO deaths, goes in alphabetical order from Argentina to USA, ending with two last deaths from Ukraine after monitoring switch from CRO to the sponsor; TRTRTXT—randomization. Treatment text; STUDYDY—Study days; NVASCLS—Sub-Classification of Non-vascular death Code (12); **—entries 787 and 788 belong to ticagrelor patients.

## Data Availability

Data are the property of the authors and can become available by contacting the corresponding author.
